# 基于新污染物微塑料检测探索仪器分析中色谱光谱融合教学方法

**DOI:** 10.3724/SP.J.1123.2025.04035

**Published:** 2025-08-08

**Authors:** Shudi LIU

**Affiliations:** 烟台大学化学化工学院，山东 烟台 264005; College of Chemistry and Chemical Engineering，Yantai University，Yantai 264005，China

**Keywords:** 色谱, 光谱, 综合教学, 海洋新污染物, 微塑料, chromatography, spectroscopy, integrated instruction, new marine pollutants, microplastics

## Abstract

仪器分析课程属于大学本科基础课程，主要面向化学、材料、生物、环境及食品等专业。其中，色谱和光谱是仪器分析课程中的重点教学内容，理论教学体系及配套的教学实验比较成熟。然而，课程各个章节中的知识点繁杂、联系不紧密，学生在基础理论知识和实验技术方面进行拓展与综合运用较难，在创新性和前沿性方面还相对欠缺。本文利用沿海学校的地理优势，基于海洋新污染物微塑料的检测案例探索色谱与光谱的融合教学方法，从教学内容、教学模式及教学效果方面探讨了微塑料的分析进展。开展本课程不仅可以引入热点话题拓宽学科知识面，而且能够提升学生调研相关文献、了解科学前沿发展、掌握仪器综合运用的能力，培养学生的创新意识和科学素养，最终实现立德树人的根本任务。

## Abstract (en)

Instrumental analysis is a basic undergraduate course. It is mainly teaching-oriented for chemistry， materials， biology， environment， food and other majors. Among them， chromatography and spectrometry are two of the key teaching contents in the course of instrumental analysis. They have mature theoretical teaching systems and the supporting teaching experiments are relatively coincident. However， the main knowledge points are cluttered and complicated， and the connections between these chapters are not close enough. It’s hard for students to expand and comprehensively apply knowledge on the basis of theoretical knowledge and experimental technology. Besides， the innovation and cutting-edge aspects are relatively lacking. Based on the geographical advantages of coastal schools， this paper combined with the cases of new pollutants microplastics to explore the integrated teaching method of chromatography and spectroscopy in instrumental analysis. It systematically expands the teaching content， teaching mode and teaching effect respectively. The teaching contents of chromatographic and spectroscopic principles are expanded. The analysis abilities were demonstrated by case teaching methods and comparative teaching method. The integrated method of chromatography and spectroscopy can combine the characteristics to achieve the effective complementarity of the two methods. It combines the fast high-throughput analysis abilities of chromatography and the high selectivity and sensitivity of spectroscopy. This course is conducive to promoting the standardization and normalization of screening and identification methods for new pollutants. It can provide more advanced analytical techniques for monitoring new pollutants. In addition， this course can introduce hot topics to broaden the scope of subject knowledge. It is helpful to improve students’ ability to investigate relevant literature， understand scientific frontier development of science， and master the comprehensive application of instruments. It is also beneficial to guide students to master the induction， comparison and analysis of basic theoretical knowledge， which is conducive to students’ integration of instrument analysis. At the same time， it helps students grasp the bottleneck problems in the process of the instrument development. It then motivates students to become leading talents to meet the major strategic needs of the country. This paper is in favor of cultivating students’ innovative awareness and scientific spirit， and finally realizing the fundamental task of establishing morality and cultivating talents.

仪器分析课程属于大学本科基础课程，主要面向化学、材料、生物、环境及食品等专业。传统仪器分析教学以培养学生的基础理论知识、熟悉仪器结构单元及定性定量分析为主要目标，配套的教学实验以巩固基本理论知识为主要目标，属于验证性实验，创新实验及综合性实验涉及较少。另外，课程各章节知识点繁杂、联系不紧密，学生在基础理论知识和实验技术方面进行拓展与综合运用的机会较少，缺乏实际复杂样品的剖析能力。因此，仪器分析教学在培养科技创新人才方面肩负重担。培养学生综合运用多种实验技术方法，从多角度分析实验结果，并尽可能与科技前沿紧密结合，已成为当前仪器分析教学的主要任务^［1-3］^。

近年来，大型科研仪器设备在解决重大科技攻关难题和突破经济社会发展方面发挥了高效作用。但目前地方高校大型仪器的使用主要定位于科研服务，在人才培养方面只发挥了微弱的作用。因此，高校逐渐意识到当前仪器分析教学的不足，努力聚焦综合教学方法，通过多种技术的联用，并利用科研成果反哺教学的方式，实现人才培养与教学方法的快速提升^［4-7］^。色谱和光谱是仪器分析课程中的重点教学内容，理论教学体系及配套的教学实验比较成熟，但为了进一步提高教学效果，实现教学改革与人才培养，设计相关的综合性实验也是当前教学的发展趋势^［8］^。色谱与光谱在新污染物的识别与筛查中扮演了非常重要的角色^［9］^。例如微塑料广泛分布于江河湖海及海洋沉积物中，其形式复杂多样，含量处于痕量或超痕量水平，对分析检测提出了更高的要求。

本文以海洋新污染物微塑料的检测为案例，探索仪器分析中色谱、光谱融合教学方法，以综合教学为主要课程目标展开教学工作。将色谱技术快速高通量分析的特点与光谱技术高选择性和高灵敏度分析的优势结合，探讨新污染物的光谱与色谱分析研究进展。并进一步比较了两种方法的分析原理与识别能力，从教学内容、教学模式及教学效果方面分别阐述，以求拓展新污染物的分析检测能力，培养仪器分析领域的专业技术人才，为环境可持续发展做出积极贡献。

## 1 仪器分析教学现状

根据我校教学大纲要求，理论课程设置为48学时或32学时，通常安排在第二学年下学期或者第三学年上学期，并配套同等学时的实验教学，因而在教学过程中往往存在课时不足的问题。此外，仪器分析涉及光谱、色谱、质谱、电化学、核磁等不同分析方法，课程各个章节知识点较多却联系不紧密。且仪器分析实验主要是验证性实验，综合性不强，在创新性和前沿性方面还相对欠缺。

（1）在色谱教学过程中，需要学生掌握分离原理，熟悉仪器部件及控制条件，筛选出适宜的分离条件，并能分析各物质的分离度。配套实验主要包括液相色谱法测定混合物中苯和甲苯，毛细管色谱法测定邻、间、对二甲苯含量，以及离子色谱法测定饮用水中阴离子。教学内容主要囊括了常规化合物或离子的检测，对于新污染物的检测很少涉及。另外，在实际教学过程中采用大班理论教学-小组实验模式。在教学过程中即使结合部分视频教学，但由于时间限制，学生对原理的掌握不够深入。在分组实验过程中由于仪器数量少，资源有限，教师现场演示过程覆盖面小，导致学生的仪器操作能力大打折扣，无法满足学生对诸多控制条件的有效筛选，更不可能满足学生对于特定物质的探索性研究。

（2）在光谱教学过程中面临同样的问题。需要学生掌握光学检测原理，熟悉仪器部件的功能及控制条件，并进一步筛选出适宜的检测条件。配套实验主要包括原子吸收光谱法测定钙含量、紫外吸收光谱法测定废水中的微量酚、分子荧光光谱法测定维生素B2的含量以及未知化合物的红外光谱分析等。在实际光谱教学过程中，教学内容、教学模式及教学效果同样不能满足当下科技高度发展条件下对学生的培养需求。

## 2 综合教学设计

本文针对当前仪器分析教学中存在的具体问题，从教学内容、教学模式、教学效果3个方面展开海洋新污染物的案例分析与综合教学工作。

### 2.1 教学内容

由于塑料制品的大量使用和管理问题，目前在环境基质中都可以检测到微塑料，如地表水、海洋、深海沉积物以及各类动植物体内^［10］^。因此在理论教学过程中，尤其在色谱教学及光谱教学中，引入当前热门的新污染物微塑料进行内容拓展，从检测角度突出不同方法的侧重点。

（1）在色谱教学过程中，引入文献报道案例讲解海洋微塑料的分析方法。例如热解-气相色谱-质谱法可以实现环境样品中微塑料的鉴定^［11］^。这种方法主要采用热分析方式进行样品分析，在1 min内完成检测，微塑料样品最低检出限低于1 μg。方法充分发挥了气相色谱法所需样品量小的特点，亦可实现不同类型微塑料及相应添加剂成分的鉴定。然而这种方法的缺点在于无法获得微塑料的形貌特征，对分析样品有破坏性，对于复杂样品的研究缺乏全面性和普适性。因此，后续可以结合光谱法弥补这些不足。

（2）在光谱教学中，引入相应荧光、红外或拉曼等检测方法的案例，分析微塑料的尺寸和化学成分。例如研究发现亲脂性染料尼罗红可以实现海盐样品中50 μm微塑料颗粒的荧光检测^［12］^。这种方法操作简单，适用于快速筛选微塑料。此外，在红外、拉曼等的教学过程中亦可列举相应的检测案例，重点关注检测条件、分析特点及应用等方面。在学生充分了解所有仪器分析教学方法的特点后，引入近年来海洋微塑料检测方法的综述进行归纳总结^［13，14］^，指出各种方法在检测过程中的难点与可突破点，提高学生的科研兴趣及综合评价能力。

### 2.2 教学模式

在教学过程中还需采用适宜的教学模式达到启发学生的目的，常用案例教学法和比较教学法两种方式来进行。

#### 2.2.1 案例教学

在案例教学中，可以根据文献中的具体实施案例进行分步介绍。把握重点内容如样品前处理、检测过程、检测结果等，也涉及主要试剂或药品、仪器及设备等方面，在教学过程中对相关实验过程作简要介绍，有利于学生按照实验过程先后顺序进行准备，起到了引导学生对实验课程提前预习的作用。例如采用香豆素进行微塑料检测^［15］^，首先从海水中收集样品颗粒，进一步通过氧化处理和密度分离除去样品中残留的沙粒，再利用丙酮和乙醇等溶剂按照不同比例探究微塑料的耐溶解性能，在此情况下实现香豆素染料染色。在检测过程中通过荧光强度测试，并结合高分辨成像，实现不同种类微塑料的区别性检测。最终的检测结果将根据微塑料尺寸与荧光强度关系实现不同微塑料聚合物类型的辨别。

#### 2.2.2 比较教学

比较教学法在教学过程中也非常适用。通过比较突出各类方法的侧重点，有利于培养学生的认知能力及总结归纳能力，找出优势互补的中心点^［16］^。例如扫描电子显微镜和透射电镜法可以识别粒径低至1 nm的微塑料，但不能用于化学成分分析。荧光染色可以检测大部分微塑料，但由于各类微塑料的光谱有覆盖现象以及天然荧光物质的干扰，无法有效区别微塑料的类型。傅里叶变换红外光谱和拉曼光谱可以分析微塑料的化学成分，但在粒径和形态方面的分析不足。气相色谱和液相色谱能够准确分析微塑料及添加剂的有效成分，但需要破坏样品，同样无法满足粒径和形态分析。各种方法对微塑料的粒径、形态、类型及浓度等方面的测定侧重点不同^［17］^，可以通过列表的形式（表1），将各类方法进行比较，并进行优势互补。

**表1:** 海洋污染物微塑料的检测方法比较

Method	Size	Shape	Type	Concentration	Ref.
TEM	+	+	-	-	［^18^］
SEM	+	+	-	-	［^19^］
Py-GC-MS	-	-	+	+	［^11^］
LC-MS	-	-	+	+	［^20^］
FTIR	+	-	+	+	［^21^］
SERS	+	-	-	+	［^22^］
Fluorescence	+	+	-	+	［^23^］

TEM： transmission electron microscope； SEM： scanning electron microscope； Py-GC-MS： pyrolysis-gas chromatography-mass spectrometry； LC-MS： liquid chromatography mass spectrometry； FTIR：Fourier transform infrared spectroscopy； SERS： surface enhanced Raman scattering. +： The analysis method is available； -： The analytical method is unavailable.

#### 2.2.3 实验教学

相比于传统的色谱实验或光谱实验，综合实验教学的目的与指标涵盖了综合的实验内容以及实验方法两个方面。本文选择红外光谱与色谱联用作为教学案例，一方面满足目前教学大纲的要求；另一方面实验室关于红外光谱实验的教学设备、参考资料以及标准图谱相对充足。检测海洋微塑料的色谱光谱综合实验过程主要包含以下几个方面：（1）样品的处理。在处理微塑料样品时，首先从海水中收集样品颗粒，进一步通过氧化处理和密度分离除去沙粒，再利用碱性K_2_S_2_O_8_方法消解处理海水中的微塑料^［24，25］^，相比于传统的HCl法、NaOH法或酶解法，生物基质等杂质更少，检测效果更佳。（2）样品的测试。在测试前先进行颗粒计数分析，获得微塑料的平均粒径、质量，再将样品分为两部分同时进行测试。在光谱测定中，主要采用红外光谱法进行分析，对样品种类及浓度两个方面进行控制，准备3种常见微塑料的标准品，与测试样品进行图谱比较，获得微塑料样品的类型以及浓度。在气相色谱测定时，从样品进样器、色谱柱、柱温、检测器，尤其在流动相及流量控制方面实现指标化，获得微塑料样品的质量及成分分析。（3）实验数据分析。首先将两种方法获得的实验数据进行综合分析。由于两种方法在颗粒和质量浓度方面具有相似的趋势，证明两种方法具有良好的总体可比性，同时结合标准品的图谱得出微塑料的主要成分。其次，由于微塑料的成分与粒度相关，且粒度服从幂次分布，因此，可以将微塑料的平均粒径、质量作为参考颗粒，再将每个颗粒的面积除以参比颗粒的参比面积，再乘以参比颗粒的质量。通过计算使粒子数据的权重具有更好的一致性^［26］^。将样品中存在的不同类型、形状和大小的微塑料的颗粒组合，再经过进一步加权计算，据此确定微塑料临界质量与粒径和颗粒形状的关系。学生通过实验方案的设计，实际样品的分析检测，能够合理有效地推演实验结论，并充分了解现代仪器分析的基本方法。

#### 2.2.4 结果反馈

教学完成后进行教学评估。学生对于综合实验表现出明显的兴趣，在样品处理、仪器的条件控制与优化、实验结果分析讨论方面都有极大提升。学生还可以针对污染程度不同的微塑料样品（处理后的废水样品、海洋沉积物样品及海洋表层水样品）进行测定，根据样品的复杂程度与污染程度建模，获得相应的定性定量数据，从而进行创新性实验分析。

### 2.3 教学效果

色谱光谱融合教学在教学过程中体现出了明显的优势。首先，围绕当前科技动态，学生能够产生浓烈兴趣，进行热烈讨论，对于环境保护和仪器发展有了更加深刻的认识。光谱和色谱仪器的使用方法、实验过程及结果分析可激发学生积极性，充分调动学生思考能力和动手能力。色谱光谱融合教学也加深学生对基础理论知识的理解，在后续的气相色谱-红外光谱联用及高效液相色谱-红外光谱联用技术的学习方面更加得心应手。相较于传统的验证性实验，综合实验把色谱技术快速高通量分析的特点与光谱技术高选择性和高灵敏度分析的优势结合，在创新性及综合性方面优势显著。此外，若将实验拓展至荧光、紫外、拉曼光谱与色谱的综合教学过程中，能够进一步提升学生使用仪器的综合能力。这种融合教学对于仪器分析的整体教学有利，使教学质量和教学效果进一步改善。

其次，综合实验在时间和流程上凸显出优势。原来两个实验课程各自4学时合并为8学时，学生只需要处理同一类实验样品，大大缩短了实验前处理时间。实验过程中学生只需准备一组标准样品，且每台仪器上只需要测定一次标准曲线，减少各组标准曲线的重复次数，亦能够节省出大量的时间用于后续实验测试（图1）。学生在实验时可以分成更多小组轮流测试未知样品，由于多个实验室及仪器同时运行，避免了扎堆现象^［27］^，在实验过程中实现了学生对诸多控制条件的有效筛选，从而对于特定物质的定性定量检测及探索性研究有更充分的时间。此外，每台仪器同时运行，也缩短了两种仪器的预平衡时间。减少仪器开关机频率，还能够延长仪器的使用寿命。

**图1:**
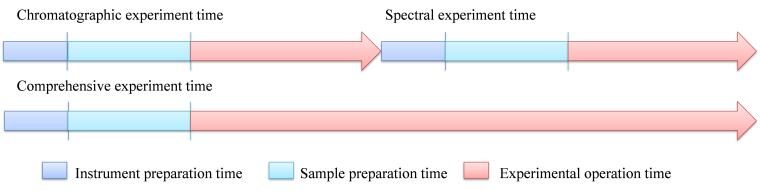
色谱光谱实验与综合实验的时间轴对比图

第三，综合实验教学与前沿科学问题的紧密结合不仅提升了学生基础理论知识拓展与综合实验技术运用的能力，也培养了学生对实验结果的多维度分析能力。同时结合科研成果反哺教学的方式，实现了教学质量的快速提升。

## 3 结论与展望

目前，我国教育及尖端仪器发展已取得较大进步，但当前仍存在国产分析仪器原创力不足的情况。同时，新污染物（如微塑料）筛查与识别方法的标准化和规范化还有待进一步加强。相较于传统筛查与识别技术，色谱光谱联用技术集高通量、高选择性、高灵敏度、自动化为一体，两种技术相互佐证并互为补充，能够拓宽新污染物定性定量识别能力，有望为新污染物监测提供更先进的分析技术。本文从培养仪器分析领域的专业人才角度出发，通过新污染物微塑料的检测案例，引导学生评价光谱与色谱两种方法对新污染物的识别与筛查能力，提升仪器分析的综合教学质量和教学效果，为拔尖人才的培养奠定基础。首先，通过综合教学不仅有利于学生掌握基础理论知识以及仪器综合运用的能力，而且可以引入热点话题拓宽学科知识面，提升学生调研相关文献、了解科学前沿发展、加强归纳总结的能力。其次，从超痕量、实时、原位、高通量、精准测量等方面的需求入手，引导学生建立相应的标准方法，实现污染物的精准检测与有效管理，是当前仪器分析教学的主要任务。第三，自动便携式监测仪器尚未发展成熟。微型化、智能化、多元一体化关键部件及核心技术发展迫切，需要培养学生精益求精的大国工匠精神，激励学生突破仪器发展过程中的“卡脖子”问题，成为解决国家重大战略需求的领军人才。最后，培养学生恪守职业道德，深刻明确国家情怀与社会责任，才能最终实现立德树人的根本任务。
